# Dietary aflatoxin exposure of lactating mothers of children 0–6 months in Makueni County, Kenya

**DOI:** 10.1111/mcn.13493

**Published:** 2023-02-22

**Authors:** Isaac O. Ogallo, Dasel W. M. Kaindi, George O. Abong, Alice M. Mwangi

**Affiliations:** ^1^ Faculty of Agriculture, Department of Food, Science, Nutrition & Technology, Applied Human Nutrition Program University of Nairobi Nairobi Kenya; ^2^ Department of International Agricultural Development, Graduate School of International Food and Agricultural Studies, Tropical Crop Science, Master Program Tokyo University of Agriculture Tokyo Japan; ^3^ Faculty of Agriculture, Department of Food, Science, Nutrition & Technology University of Nairobi Nairobi Kenya; ^4^ Udugu, Ufanisi Ustawi wa Jamii (3UJ) Development Limited Nairobi Kenya

**Keywords:** aflatoxin B1, breastfeeding children, dietary aflatoxin exposure, lactating mothers, Makueni Kenya, total aflatoxin

## Abstract

The southeastern region of Kenya is prone to aflatoxin outbreaks, yet maternal and infant aflatoxin intake levels remain unclear. We determined dietary aflatoxin exposure of 170 lactating mothers breastfeeding children aged 6 months and below in a descriptive cross‐sectional study involving aflatoxin analysis of maize‐based cooked food samples (*n* = 48). Their socioeconomic characteristics, food consumption patterns and postharvest handling of maize were determined. Aflatoxins were determined using high‐performance liquid chromatography and enzyme‐linked immunosorbent assay. Statistical analysis was conducted using Statistical Package Software for Social Sciences (SPSS version 27) and Palisade's @Risk software. About 46% of the mothers were from low‐income households, and 48.2% had not attained the basic level of education. A generally low dietary diversity was reported among 54.1% of lactating mothers. Food consumption pattern was skewed towards starchy staples. Approximately 50% never treated their maize, and at least 20% stored their maize in containers that promote aflatoxin contamination. Aflatoxin was detected in 85.4% of food samples. The mean of total aflatoxin was 97.8 μg/kg (standard deviation [*SD*], 57.7), while aflatoxin B1 was 9.0 μg/kg (*SD*, 7.7). The mean dietary intake of total aflatoxin and aflatoxin B1 was 7.6 μg/kg/b.w.t/day (*SD*, 7.5) and 0.6 (*SD*, 0.6), respectively. Dietary aflatoxin exposure of lactating mothers was high (margin of exposure < 10,000). Sociodemographic characteristics, food consumption patterns and postharvest handling of maize variably influenced dietary aflatoxin exposure of the mothers. The high prevalence and presence of aflatoxin in foods of lactating mothers are a public health concern and calls for the need to devise easy‐to‐use household food safety and monitoring measures in the study area.

## Key messages

- A high prevalence of aflatoxin (84.4%) and high dietary aflatoxin exposure were reported among lactating mothers in the study.
- The mean concentration of 90% of maize‐based cooked food samples exceeded the 2 and 10 µg/kg set limits for aflatoxin B1 and total aflatoxin, respectively.
- Household size, education, socioeconomic status and limited food consumption patterns were potential determinants of dietary aflatoxin exposure in lactating mothers. Within dietary diversity, the type of food constituting a diverse diet played an important role.
- Postharvest handling of maize could be one of the factors contributing to dietary aflatoxin exposure in the study area.
- The reported results call for the need to devise easy‐to‐use household food safety and monitoring measures in the study area.

## 1 INTRODUCTION

Aflatoxins are secondary metabolites of fungal origin. They are released as spores that can withstand a range of extreme environmental conditions (Kumar et al., 2021). Their occurrence around the globe depends on geographic, climatic, agronomic and agricultural factors (Mahato et al., 2019). They can enter foods pre or postharvest (A. Kumar et al., 2021), and subsequently be consumed by humans and animals. Due to their harmful effects on animal and human health, they are intensively studied (Akbar et al., 2019).

The first incidence of aflatoxins' potency was reported in 1960 upon the death of 10,000 Turkeys and ducklings in the United Kingdom (Bhat et al., 2010; Blount, 1961). Since then, several incidences of aflatoxin contamination have been reported worldwide (Pickova et al., 2021). In Kenya, the first fatal case was reported in 1981 in the southeastern region of the country (Machakos, Kitui, Mwingi and Makueni former districts) (Omara et al., 2021). Since then, several fatalities and frequent episodes of aflatoxin contamination have been reported in the country (Mutegi et al., 2018; Omara et al., 2021). To date, alerts of aflatoxin‐contaminated maize still feature in Kenya's news. In Nying'uro's (2020) study, incidences of aflatoxin occurrence in hotspot areas in Kenya are expected to increase due to climate changes.

Among aflatoxin studies examined by Omara et al. (2021), maize and other foods, mainly cereals, animal milk and animal feeds, were given more focus in Kenya. Additionally, it was observed that samples collected from the southeastern region of Kenya were associated with higher levels of aflatoxin compared to samples from other regions. However, among the few human studies, serum and urine were given more focus, whereas breast milk was only analysed by Kang'ethe et al. (2017) and Maxwell et al. (1989). Similarly, the studies that estimated exposure of lactating mothers to dietary aflatoxins were also lacking, except for a study conducted by Obade et al. (2021) among pregnant mothers in Kisumu County, the western region of Kenya. From this, it is clear that exposure of lactating mothers was not given more focus in Kenya, yet it could be one of the issues that require public health attention in the study region. This is because studies, including that of Kumar et al. (2016) among others, have associated high levels of aflatoxin exposure with acute aflatoxicosis characterised by haemorrhage, oedema and acute liver damage, while prolonged intakes, on the other hand, in small doses, have been shown to cause chronic aflatoxicosis, cancer, birth abnormalities in the foetus, malnutrition and immune suppression. That notwithstanding, it has also been shown that higher levels of aflatoxins in mothers' diets could lead to higher levels of aflatoxin M1 in breast milk (Mehta et al., 2021), and, consequently, have a higher negative health effect on breastfeeding children compared to adults (A. Kumar et al., 2021). Additionally, this could also have an impact on the quality of breast milk that children aged 6 months and below solely depend on. According to Boquien (2018), breast milk is important during the first 6 months of life, is considered safe and acts as the primary source of nutrition for the young ones before they can eat other foods. Compromised breast milk safety could thus be one of the factors that could cause serious challenges in improving rates of exclusive breastfeeding in Kenya for children aged 6 months and below. However, at the time of conducting this study, information regarding the aflatoxin exposure of lactating mothers breastfeeding children of this age category remains scanty.

To reduce the negative impact associated with aflatoxin, it is important to determine the magnitude and reduce the risk of exposure. However, the government's effort to ensure the implementation of food safety and control measures for aflatoxins in Kenya still faces several challenges, especially among small and medium enterprises dealing with maize (Joutsjoki & Korhonen, 2021). These challenges could still pose a threat to ensuring that lactating mothers are not exposed to aflatoxins through their diet. As a result, this study sought to determine dietary aflatoxin exposure of lactating mothers of children aged 6 months and below in Makueni County, Kenya. However, as aflatoxin analysis in breast milk was included in the larger part of this study, dietary aflatoxin exposure of mothers was also determined based on breastfeeding status.

## 2 METHODS

### 2.1 Sampling procedure

This study was part of a larger descriptive cross‐sectional study with an analytical component conducted among lactating mother–child dyads in the Kikumbulyu location, Kibwezi‐West Constituency, Makueni County, Kenya. A multistage sampling procedure was used to select the targeted households. The sample size of lactating mothers in the study was determined using Fisher et al.'s (1991) formula (*n* = *Z* ^2^ *pq*/*d* ^2^) where *n* is the desired minimum sample size; *Z* is the standard normal deviation set as 1.96 corresponding to 95% confidence interval (CI); *p* is the prevalence of aflatoxins maize samples above 10 µg/kg estimated at 87.3% in Makueni (IFPRI, 2010); *q* = 1 − *p* (proportion of maize sampled without aflatoxins), that is, 0.127; and *d* is the degree of accuracy set as 5%, that is, 0.05. A sample size of 170 lactating mothers was arrived at as shown in the calculation:

$$\begin{array}{l} n=\left(1.96^{2}\times 0.873\times 0.127\right)/\left(0.05\times 0.05\right)\\ \;=170.36\\ \;\approx \;170. \end{array}$$

Makueni County was selected based on several aflatoxin contamination incidences reported in the area, while Kibwezi Sub‐County was selected due to its dense population compared to other sub‐counties (Kenya Population and Housing Census [KPHC], 2019a, 2019c). Kibwezi West Constituency was selected following high aflatoxin results by Kilonzo et al. (2014) in the area. Kikumbulyu location was preferred because it had a higher proportion of children aged between 0 and 5 years compared to other sublocations (KNBS & SID, 2013).

Mothers of reproductive age (15–45 years), having a child aged 6 months and below, and ideally, should be lactating at the time of conducting the survey were included in the study based on their availability and willingness to participate. The mothers who did not meet these criteria were excluded from the study. The households with the targeted mothers were identified using the Expanded Program Immunization (EPI) coverage random walk method adopted by World Health Organization (2008). With the assistance of local guides in the study area, a central point was identified and the starting point was determined by spinning a bottle. However, access paths were followed to avoid leaving out remote dwelling units as opposed to main roads. In the case of two paths, a coin was flipped once, while in cases where there were more than two paths, a coin was flipped several times until a decision was made. The process was repeated until the desired sample size of 170 was achieved.

The number of foods to be sampled in the study was also determined using Fisher et al.'s (1991) formula (*n* = *Z* ^2^ *pq*/*d* ^2^) where *n* is the desired minimum sample size; *Z* is the standard normal deviation set as 1.96 corresponding to 95% CI; *p* is the prevalence of malnutrition (<−2 standard deviation [SD] weight‐for‐height for children below 5 years, Makueni) estimated at 2.1% (KDHS, 2014); *q* = 1 − *p* (proportion of children not <−2 SD weight‐for‐height), that is, 0.979; and *d* is the degree of accuracy set as 5%, that is, 0.05. An arbitrary attrition rate of 0.2 was added to take into account the anticipated challenges of obtaining cooked maize‐based food samples at the time of the survey. A sample size of 40 foods was generated as shown.

$$\begin{array}{l} n=\left(1.96^{2}\times 0.021\times 0.979\right)/\left(0.05\times 0.05\right)\\ \;=31.6\\ \;=31.6/\left(1-0.2\right)\\ \;=39.5\\ \;\approx \;40. \end{array}$$

This present study targeted cooked maize‐based food samples that were prepared and consumed by lactating mothers but remained or left for subsequent consumption at the time of data collection. To meet the calculated sample size (*n* = 40), at least five cooked maize‐based food samples were purposively targeted per each of the eight wards in the study area. Any maize‐based food sample was collected per household as long as it was consumed by the targeted mother. However, the collection of food samples was based on chance (i.e., if maize‐based food remained after a meal, and availability and the willingness of a lactating mother to give out the food samples during the EPI random walk and administration of study questionnaires). Maize‐based foods were purposively sampled in the study due to their high susceptibility to aflatoxin contamination.

### 2.2 Ethical consideration

Ethical issues at all stages were considered in the study by obtaining ethical clearance (P454/08/2013) from Kenyatta National Hospital/The University of Nairobi‐Ethical Review Committee (KNH/UoN‐ERC). After approval of the research by the ethical committee, a meeting and discussion were held with the administrative and local community leaders, the ministry of health in charge and community health workers to seek permission before conducting the study in the area. This was followed by subsequent awareness of the study at the village level whose aim was to target lactating mothers and their spouses. A joint briefing was conducted where issues about the study safety, objectives, voluntary participation and confidentiality of the study participants were highlighted before administering the study questionnaires. During data collection, mothers who were willing to participate in the study signed informed consent. However, mothers were also at liberty to discontinue participating in the study even after giving consent.

### 2.3 Data collection

#### 2.3.1 Sociodemographic and economic status of lactating mothers

A pretested semistructured questionnaire was administered to collect information on the socio‐demographic characteristics of the lactating mothers (age, marital status, household size, number of children, area of residence, occupation, education level, asset ownership, income, savings and expenditure). The aggregate economic status of lactating mothers in the study was constructed using the principal component analysis method (KDHS, 2014). Lactating mothers were then grouped into lower (total score ≤ 9), middle (total score 10–19) and upper wealth index (total score ≥ 20).

#### 2.3.2 Consumption frequency of foods

Food frequency targeting foods that are consumed on a weekly basis was determined using a pretested semiquantitative frequency questionnaire. Consumption quantities of foods that are likely to be contaminated by aflatoxin were determined using household measures and a food atlas compiled by Ojwang‐Ndong (2013). Daily estimated quantities were arrived at using the formula:

Estimated daily consumption quantities=(Q×F1×F2)/7,

where *Q* is the estimated quantities of food consumed per sitting (g), *F*1 is the frequency of consumption within a typical day, *F*2 is the frequency of consumption per week, and 7 is the reference period of consumption frequency. Foods that were rarely consumed, for instance, once a month, were left out. It was also assumed that quantities of food were equally spread within a week in this calculation. Questions regarding the main source of maize, postharvest handling and storage of maize were also collected during the survey.

#### 2.3.3 Aflatoxin‐prone foods weekly consumption score of each lactating mother

The weekly consumption frequency score mainly for 18 selected foods that are likely to be contaminated by aflatoxins was determined (Supporting Information: Appendix 1). Foods that were consumed daily were given a weight of seven while those that were consumed twice, thrice or four times in that order until six times per week were given weights of two, three, four, five and six, respectively. A weight of zero was given to foods consumed per 2 weeks, per month and never as shown in Supporting Information: Appendix 1. The weights were multiplied by their respective consumption frequencies reported within a day to generate a total weekly consumption score as shown in the formula:

$$\begin{array}{l} \text{Total weekly consumption score for aflatoxin}-\text{prone foods}\\ =\text{Assigned food consumption weight}\times \text{consumption frequency within a day.} \end{array}$$

The weighted aflatoxin consumption score for each lactating mother was subsequently derived by summing up the total weekly consumption score of all aflatoxin‐categorised foods reported by the mothers and dividing the summed total consumed with a denominator of 504. The denominator was arrived at by multiplying the expected maximum food score per week (7) of each food by the expected maximum frequency consumption within a day (4) by the total number of foods (18) listed as foods likely to be contaminated by aflatoxins in this present study and expressing the result to a percentage shown as follows:

$$\begin{array}{l} \text{Weighted aflatoxin consumption score per lactating mother}\\ =\left(\sum \left[\text{score food }1+\text{score food }2+\text{…}+\text{score food }18\right]\times 100\%\right)/504. \end{array}$$

Lactating mothers were further categorised into quartiles according to their percentage scores.

#### 2.3.4 Dietary diversity

A guideline for measuring dietary diversity (FAO, 2011) was used to generate 24‐h dietary diversity scores for lactating mothers. The score of 13 food groups (cereals, roots and tubers, vitamin A‐rich vegetables and tubers, dark green leafy vegetables, other vegetables, vitamin A‐rich fruits, other fruits, organ meat, flesh meats, eggs, fish and seafood, legumes, nuts and seeds, milk and milk products) were aggregated into 9 food groups where cereals and white tubers and roots were combined into starchy staples, other vitamin A‐rich fruits and vegetables formed one group, other fruits, and other vegetables formed another group, and meat and fish formed a single group to generate women's dietary diversity score^3^ (WDDS). Lactating mothers who had WDDS ranging between 1 and 3 were categorised as mothers with low dietary diversity, while the ones with scores between 4 and 6 and 7 and 9 were categorised as mothers with medium and high dietary diversity, respectively.

#### 2.3.5 Food samples

Solid food samples were collected using the quartering method (Campos‐M & Campos‐C, 2017). A representative sample was drawn from the top, middle and bottom of a plate, bowl or cup. Semiliquid foods were stirred to mix and a representative sample was scooped from the middle. The samples were transferred into a weighed cup until a 60 g sample weight was attained (50 g for quantification and 10 g for detection). The samples were stored in an ice‐filled cooler box at temperatures of 4°C for 3 days (restocking of ice was done daily at the end of every data collection day) before being transferred to a deep freezer at −18°C. Food samples collected were solid maize meal (ugali, *n* = 18), semiliquid maize porridge (*n* = 6), maize‐sorghum porridge (*n* = 9) and a mixture of boiled maize and beans (githeri, *n* = 9) and a mixture of dehulled maize and beans (muthokoi, *n* = 6). The varying proportion of food samples was a result of picking foods that remained after the household had had their specific meal at the time of data collection.

### 2.4 Analytical methods

#### 2.4.1 Detection of positive aflatoxin in cooked maize‐based food samples

Aflatoxin was detected as trifluoracetic derivatives using high‐performance liquid chromatography (HPLC) and fluorescence detector (Nexera X2 Model; Shimadzu). For this process, 5 g of the samples were ground to fineness. Extraction was done using 25 mL of 70% methanol. The cleaning‐up procedure was done using Romer AflarStar‐immunoaffinity column, while aflatoxin derivatization was done using 200 µL trifluoracetic acid. Reverse‐phase HPLC column (Lichrospher® RP‐18, 250 × 4.0 mm I.D., 5 µm) was used for separation. Identification was done at a run time of 30 min, a velocity of 1.0 mL/min, an injection volume of 10 µL, a column temperature of 35°C, an excitation wavelength of 363 nm, an emission wavelength of 440 nm and sloppiness of 10 nm using a fluorescence detector.

#### 2.4.2 Determination of total aflatoxin and aflatoxin B1 in positive food samples

Positive samples were quantified for total aflatoxin and aflatoxin B1 using Ridascreen ELISA competitive enzyme immunoassay (r‐Biopharm) with slight modification. Samples collected (50 g) were ground and mixed with 250 mL methanol–water mixture (70%:30%, v/v) and homogenised for 3 min for extraction. The resulting solution was filtered using Whatman filter paper number 1, and 50 µL was used for each standard and sample per well. Provided conjugate and antibody (50 µL each, respectively) were added, and incubation was done for 30 min at room temperature (25°C). A wash buffer (phosphate buffer with tween) of 250 µL was used. Chromogen (100 µL) was added as substrate and incubated again for 15 min at room temperature. Acid stop solution was added and the reading was done within 15 min. The recovery rate was set at 85% for total aflatoxin, and 93% for aflatoxin B1, while the absorbance reading was determined at 450 nm. However, the lower detection limit for total aflatoxin and aflatoxin B1 was set at 1.75 and 0.5 µg/kg, respectively.

#### 2.4.3 Determination of dietary aflatoxin intake of lactating mothers

Dietary aflatoxin intake was determined as shown in the formula:

$$\text{Aflatoxin intake }\left(µg/\mathrm{kg}/\mathrm{kg}b.w.t/\mathrm{day}\right)=\frac{\text{Aflatoxin concentration}\left(µg/\mathrm{kg}\right)\times \text{Estimate quantities of food consumed }\left(g\right)/\mathrm{day}}{\text{Body weight of lactating mother }\left(b.w.t\right)\left(\mathrm{kg}\right)\text{.}}$$

The contribution of each analysed food on the cumulative total aflatoxin and aflatoxin B1 intake in the study was determined based on regression coefficients derived by running the @Risk simulation model using the mean and range of aflatoxin intake for each food, risk β general distribution and 10,000 iterations.

$$\text{Aflatoxin intake}\left(µg/\mathrm{kg}/\mathrm{kg}b.w.t/\mathrm{day}\right)=\frac{\text{Aflatoxin concentration }\left(µg/\mathrm{kg}\right)\times \text{Estimate quantities of food consumed }\left(g\right)/\mathrm{day}}{\text{Body weight of lactating mother }\left(b.w.t\right)\left(\mathrm{kg}\right)\text{.}}$$

#### 2.4.4 Determination of margin of exposure (MOE) of lactating mothers to dietary aflatoxin intake

The MOE was derived by taking the benchmark dose level (BMDL) of total aflatoxin and aflatoxin B1 and dividing it by the estimated mean and 95th percentile of aflatoxin intake of lactating mothers in the study (European Food Safety Authority [EFSA], 2005). Benchmark dose (BMD_10_) of 0.41 μg/kg/b.w.t/day adequate to increase tumour by 10% in male rats (EFSA CONTAM Panel et al., 2020) was used as illustrated in the formula:

$$\text{The margin of exposure }\left(\mathrm{MOE}\right)=\frac{\left(\mathrm{BMDL}\right)\text{for aflatoxin }\left(0.41\right)(\mathrm{mg}/\mathrm{kg}/b.w.t/\mathrm{day})}{\text{Estimated aflatoxin intake }(\mathrm{mg}/\mathrm{kg}/b.w.t/\mathrm{day})}.$$

The risk levels of lactating mothers to aflatoxin exposure in the study depended on the MOE results. According to EFSA (2005), a calculated MOE below 10,000 in this study indicated a potential risk of lactating mothers to dietary aflatoxin exposure. On the other hand, a calculated MOE above 10,000 indicated the absence of any risk of lactating mothers in the study to dietary aflatoxin exposure. However, when the MOE was below 10,000, a value extreme from <10,000 indicated a higher risk of dietary aflatoxin exposure and a closer value to <10,000 indicated a lesser risk of dietary aflatoxin exposure in the study.

### 2.5 Statistical analyses

Data were analysed using Statistical Package Software for Social Sciences (SPSS version 27). Descriptive statistical analysis was done on socio‐demographic variables, food frequency, dietary diversity, aflatoxin food score, consumption levels and aflatoxin levels. Statistical difference between groups was determined using Student's *t*‐test (*t*) for normally distributed data, and Mann–Whitney *U* for nonnormally distributed data. Statistical difference between more than three groups was determined using the analysis of variance (ANOVA) (*F*‐test) for normally distributed data and the Kruskal–Wallis *H*‐test for nonnormally distributed data. Bonferroni *χ* ^2^ post hoc test was used for multiple pair comparisons of ranked variables, while Tukey's Kramer was used for the post hoc ANOVA test. Pearson (*r*), Kendall tau‐b (*t*_*b*_) and Spearman (*ρ*) were used to determine the correlation between normal continuous, nonnormal continuous and ranked variables, respectively, while Chi‐square (*χ* ^2^ test) was used to determine the association between categorical variables. Simple and multiple linear regressions were used for determining significant predictors of aflatoxin concentration levels in analysed foods. The significant level was set at *p* < 0.05. Palisade's @Risk software version 8.2 was also used to determine the regression coefficient of each food on cumulative total aflatoxin and aflatoxin B1 intake in the study.

## 3 RESULTS

### 3.1 Sociodemographic and economic characteristics of the study subjects

The mean household size of lactating mothers was 6.2 (*SD*, 1.3), while the mean number of children was 3.0 (*SD*, 1.7). About 90% of the lactating mothers interviewed (*n* = 170) were in the age category of 20 to 39 years with a mean age of 29.5 (*SD*,5.9) years (Table 2a, 2b). Results also showed that slightly more than 50% had satisfactorily attained the basic level of education. However, over half (52.4%) of the lactating mothers in the study were housewives, 19.4% were casual labourers and 15.9% were self‐employed. Those who depended on farming were 15.9%, while the rest (2.4%) depended on salaried employment as their main occupational status. However, occupational status was significantly different between breastfeeding groups of lactating mothers (Fisher exact, sig. two‐sided = 11.629, *p* = 0.018). Post hoc analysis using Bonferroni χ^2^ for pair comparison showed that the number of exclusively lactating mothers (30.7%) working as casual labourers was thrice that of nonexclusively breastfeeding mothers (10.5%) (*p* = 0.00). Nonetheless, over half (53.6%) of lactating mothers had a monthly income of ≤USD 75, while the rest (46.4%) had a monthly income of >USD 75 (1 US Dollar ≈ 100 Kenya shillings). The mean monthly income was USD 70.04 (SD, 18.64), while the mode was USD 80. The consumption expenditure of 59.4% of lactating mothers was ≤USD 34.40, while the monthly savings of 72.4% of the lactating mothers were <USD 10. Consequently, 49.4% of lactating mothers were in the lower wealth index, 45.3% in the medium wealth index, and only 5.3% were in the upper wealth index (Table 1).

**Table 1:** Sociodemographic and economic characteristics of lactating mothers in Kibwezi West.

Characteristics	*N* = 170 (%)	EBF (*n* = 75) (%)	NEBF (*n* = 95) (%)	Sig. (*χ* ^2^)
Household size, mean (SD)	6.2 (1.3)	6.1 (1.3)	6.3 (1.3)	
Number of children, mean (SD)	3.0 (1.7)	2.9 (1.5)	3.1 (1.6)	
Age (years), mean (SD)	29.5 (5.9)	29.6 (5.5)	29.4 (6.2)	
Age categories (years)				
15–19	4.7	4.0	5.3	0.591
20–29	46.5	45.3	47.4	
30–39	42.4	46.7	42.4	
40–49	6.5	4.0	6.5	
Education level				
No formal education	13.5	6.7	18.9	0.001*
Attempted primary	35.3	53.3	21.1	
Completed primary	28.2	21.3	33.7	
Attempted secondary	10.6	9.3	11.6	
Completed secondary	10.0	6.7	12.6	
College/university	2.4	2.7	2.1	
Main occupation				
Salaried employed	2.4	1.3	3.2	0.018*
Farmer	10.0	9.3	10.5	
Self‐employed	15.9	12.0	18.9	
Casual labourer	19.4	30.7	10.5	
Housewife	52.4	46.7	52.4	
Monthly income categories (USD^a^)				
0–25	0.6	1.3	0	0.363
>25–50	20.6	19.2	22.0	
>50–75	32.4	28.0	36.8	
>75–105	41.2	44.7	37.7	
>105–130	3.0	2.8	3.2	
>130–155	2.4	4.0	0.8	
Consumption expenditure (USD)				
≤34.40	59.4	62.7	58.8	0.530
>34.40	40.6	37.3	43.2	
Monthly savings categories (USD)				
<10	72.4	66.7	76.8	0.317
10–20	22.9	28.0	18.9	
>2000	4.7	5.3	4.2	
Asset possession				
Productive land	25.9	22.7	28.4	0.481
Own livestock	55.3	58.7	52.6	0.169
At least a mobile phone	57.6	58.7	56.8	0.876
Media accessory	44.7	42.7	46.3	0.645
Any means of transport	34.1	25.3	41.1	0.35
Wealth index categories				
<9 (lower wealth index)	45.3	37.3	51.6	0.099
10–19 (medium wealth index)	49.4	58.7	42.1	
≥20 (upper wealth index)	5.3	4.0	6.3	

Abbreviations: EBF, exclusively lactating mothers; NEBF, non‐exclusively lactating mothers; USD, United States Dollars.

^a^ USD: United States Dollars (1 USD ≈ 100 Kenya shillings).

* Significant at *p* < 0.05.

### 3.2 Consumption frequency of foods among lactating mothers

The consumption frequency of foods likely to be contaminated with aflatoxins in the study area is shown in Supporting Information: Table S1. Stiff solid maize flour paste ‘ugali’ and porridge were the most frequently consumed foods at least once per week by all (100%) lactating mothers. The consumption frequency of foods least susceptible to aflatoxin contamination is also shown in Supporting Information: Table S2. Out of 16 foods listed in the food frequency questionnaire, kale, cowpea leaves, cabbage, beans and pigeon peas were the most frequently consumed on a weekly basis.

#### 3.2.1 Weekly aflatoxin consumption score of lactating mothers

The mean weekly percentage aflatoxin consumption score for all lactating mothers (*n* = 170) in the study was 8.0% (*SD*, 3.3; range, 1.8%–20%). Consequently, all lactating mothers were categorised under the 1st quartile group (0% to <25% score) (Supporting Information: Table S3).

#### 3.2.2 Consumption estimates of foods likely to be contaminated with aflatoxin in the study

The mean quantity (g/day) of maize porridge consumed by lactating mothers (*n* = 170) was 412.3 (*SD*, 172.5; range, 105.4–861.4), while that of maize ugali was 340.5 (*SD*, 154.4; range, 107.1–720.0). Consumption quantities of maize‐sorghum porridge, ‘githeri’, ‘muthokoi’, cassava, finger millet, rice and groundnuts were less than 100 g/day. Consumption quantities of animal‐based foods were less than 20 g/day (Table 2a).

**Table 2a:** Consumption estimates of foods likely to be contaminated with aflatoxin in Kibwezi West.

	All	EBF	NEBF	
Foods	x̅ (*SD*) g/day (range)	x̅ (*SD*) g/day (range)	x̅ (*SD*) g/day (range*)*	Mann–Whitney *U* EBF* NEBF
Maize porridge	412.3 (172.5), *n* = 170	404.5 (172.6), *n* = 75	418.5 (173.0), *n* = 95	0.58
	(105.4–861.4)	(115.7–790.7)	(105.4–861.4)	
Maize *ugali* a	340.5 (154.4), *n* = 170	319.7 (130.4), *n* = 75	356.9 (169.9), *n* = 95	0.30
	(107.1–720.0)	(107.1–720.0)	(107.1–720)	
Maize sorghum porridge	59.0 (146.3), *n* = 170	67.7 (155.4), *n* = 75	52.2 (139.1), *n* = 73	0.90
	(0.0–698.1)	(0.0–655.7)	(0.0–698.1)	
*Githeri* b	93.6 (52.7), *n* = 111	90.0 (48.5), *n* = 48	96.3 (55.9), *n* = 63	0.70
	(23.1–215.2)	(23.1–211.8)	(23.1–215.2)	
*Muthokoi* ^c^	36.2 (45.9), *n* = 154	33.9 (41.3), *n* = 69	38.0 (49.4), *n* = 85	0.90
	(0.0–151.0)	(0.0–149.3)	(0–151.0)	
Finger millet	15.9 (65.7), *n* = 170	26.4 (90.3), *n* = 75	7.6 (34.6), *n* = 95	0.11
	(0.0–500.0)	(0.0–500.0)	(0–200.0)	
Cassava	38.0 (85.5), *n* = 170	37.5 (74.1), *n* = 75	38.4 (94.0), *n* = 95	0.78
	(0.0–494.0)	(0.0–247.0)	(0–494.0)	
Rice	30.6 (53.8), *n* = 170	40.9 (67.3), *n* = 75	22.5 (38.5), *n* = 95	0.07
	(0.0–327.9)	(0.0–327.9)	(0–229.5)	
Groundnuts	30.9 (23.8), *n* = 130	30.9 (20.9), *n* = 62	31.0 (26.3), *n* = 68	0.67
	(0.0–85.7)	(0.0–85.7)	(0–85.7)	
Beef	3.4 (5.2), *n* = 147	3.7 (5.5), *n* = 63	3.2 (5.0), *n* = 84	0.63
	(0.0–17.1)	(0.0–17.1)	0.0–17.1	
Chicken	0.0, *n* = 130	0.0, *n* = 57	0.0, *n* = 73	1.00
	(0.0–0.0)	(0.0–0.0)	(0.0–0.0)	
Eggs	1.7 (3.0), *n* = 151	2.1 (3.2), *n* = 69	1.4 (2.7), *n* = 82	0.22
	(0.0–7.8)	(0.0–7.8)	(0–7.81)	
Fish	0.0, *n* = 155	0.0, *n* = 64	0.0(0.0), *n* = 91	1.00
	(0.0–0.0)	(0.0–0.0)	(0.0–0.0)	
Milk tea	19.5 (27.3), *n* = 132	19.8 (27.0), *n* = 53	19.3 (27.6), *n* = 70	0.79
	(0.0–85.7)	(0.0–85.7)	(0–85.7)	

Abbreviations: EBF, exclusive breastfeeding mothers; NEBF, nonexclusive breastfeeding mothers.

^a^ *Ugali*: stiff solid maize flour paste.

^b^ *Githeri*: maize grains boiled together with beans.

^c^ *Muthokoi*: dehulled maize boiled together with beans.

* Significant at *p* < 0.05.

#### 3.2.3 Dietary diversity of lactating mothers

The mean women's dietary diversity score in the study was 3.4 (*SD*, 1.5; range 1–6). More than half (54.1%) of lactating mothers were within lower dietary diversity (1–3), while the rest (45.9%) were within the medium dietary diversity (4–6). Further analysis showed that starchy staple food groups were consumed in the preceding day by all (100%) lactating mothers in the study, followed by milk (goat/cow) (57.1%), and other fruits and vegetables (54.7%). The Legume food group was consumed by 36.5% of the lactating mothers. Vitamin A‐rich fruits and vegetables, and fish and meat food groups were both consumed by 30.6% of the mothers. Eggs were consumed by 14.1%, while organ meat was the least consumed by 2.9%.

### 3.3 Source, storage and processing of cereals by lactating mothers

Slightly more than half (52.4%) of the lactating mothers obtained their cereals mainly from the market, 40.0% cultivated, while 7.6% depended on other sources (gifts/donations/reliefs) (Table 2b). Almost half of the mothers (44.7%) did nothing to their grains during storage, while a few (5.3%) dried their cereals in the sun. Only 7.6% sorted out discoloured and disfigured grains before storage. Among those who stored their grains for a long time, 20.6% reported applying chemicals, while 12.4% mixed grains with ash before storage. The main storage containers used by mothers included sacks (37.1%), buckets (24.7%), granaries (22.4%), and other forms of bags (15.9%) (Table 2b). Before cooking grains, all mothers reported sorting out damaged grains, while 53.5% and 46.5% were washed in normal and ash‐diluted water, respectively. Those who dried in the sun before cooking were 25.3%. All the mothers mentioned sorting out particularly maize before milling (Table 2b).

**Table 2b:** Source, storage and processing of cereals by lactating mothers in Kibwezi West.

	All (*n* = 170) (%)	EBF (*n* = 75) (%)	NEBF (*n* = 75) (%)
*Major source of cereals*			
Market	52.4	49.3	54.7
Gifts/donations/relief	7.6	5.4	9.5
Shamba	40.0	45.3	35.8
*Handling cereals before storage*			
Nothing	44.7	57.3	34.7
Spray/treatment	20.6	17.3	23.2
Drying	5.3	6.7	4.2
Sorting	7.1	6.7	7.4
Cleaning	10.0	8.0	11.6
Ash	12.4	4.0	18.9
*Main cereal storage*			
Granary	22.4	20.0	24.2
Sacks	37.	41.3	33.7
Bucket	24.7	22.7	26.3
Polyethene bags	15.9	16.0	15.8
*Handling of cereals before cooking*			
Sorting	100.0	100.0	100.0
Normal water	53.5	53.3	53.7
Ash water	46.5	46.7	46.3
Dried in the sun	25.3	32.0	20.0
Sorting before milling	100.0	100.0	100.0

Abbreviations: EBF, exclusive breastfeeding mothers; NEBF, nonexclusive breastfeeding mothers.

### 3.4 Concentration levels of total aflatoxin and aflatoxin B1 in the study

Aflatoxin was detected in 85.4% (41/48) of food samples. An overall mean concentration of 97.8 μg/kg (*SD*, 57.7; range, 2.3–210.0) and 9.0 μg/kg (*SD*, 7.7; range, 0.7–32.3) for total aflatoxin and aflatoxin B1, respectively, was reported. Concentration levels of total aflatoxin and aflatoxin B1 of the analysed maize‐based foods are summarised in Table 3. Of the positive maize‐based food samples (*n* = 41), 90.2% were above the 10 μg/kg European Union (EU) limits (EU, 2010) adopted by the Kenya Bureau of Standards (KEBS) as the maximum tolerable limit for total aflatoxin. Similarly, 92.7% of the same maize‐based food samples were above the 2 μg/kg EU limits (EU, 2010) set by the EU as the maximum aflatoxin B1 tolerable limits for ready‐to‐eat maize foods.

**Table 3:** Concentration levels of total aflatoxin and aflatoxin B1 in maize‐based cooked food samples in Kibwezi West.

Total aflatoxin concentration (μg/kg)						
	All mothers		EBF mothers		NEBF mothers	
Food	x̅ (*SD*)	Range	x̅ (*SD*)	Range	x̅ (*SD*)	Range
Maize *ugali* a	109.3 (47.6), *n* = 14	38.4–168	126.4 (37.7), *n* = 8	49.5–168.3	86.7 (53.1), *n* = 6	38.4–162.8
M. porridge	48.0 (64.3), *n* = 6	2.3–173	45.5, *n* = 1	45.5	48.5 (71.9), *n* = 5	2.3–172.9
M. s. porridge	75.9 (53.4), *n* = 7	2.7–139	127.5 (16.9), *n* = 2	115.5–139.4	55.2 (48.4), *n* = *5*	2.7–112.0
*Githeri* b	130.1 (57.9), *n* = 8	60.8–210	92.7 (31.7), *n* = 5	60.8–129.5	192.4 (20.4), *n* = 3	170–210.0
*Muthokoi* c	102.9 (52.9), *n* = 6	52.5–195	135.2 (54.6), *n* = 3	88.4–195.2	70.6 (29.8), *n* = 3	52.5–105.0
All foods	97.8 (57.7), *n* = 41	2.3–210.0	114.8 (40.9), *n* = 19	45.5–195.2	83.1 (66.5), *n* = 22	2.3–210.0

**Aflatoxin B1 concentration (μg/kg)**						
	**All mothers**		**EBF mothers**		**NEBF mothers**	
**Food**	**x̅ (** ***SD*** **)**	**Range**	**x̅ (** ***SD*** **)**	**Range**	**x̅ (** ***SD*** **)**	**Range**
Maize *ugali*	12.1 (8.6), *n* = 14	2.9–32.3	10.1 (6.9), *n* = 8	2.9–21.0	14.7 (10.5), *n* = 6	4.2–32.3
M. porridge	8.9 (10.4), *n* = 6	2.7–29.9	29.9, *n* = 1	29.9	4.7 (1.9), *n* = 5	2.7–7.4
M.s. porridge	6.9 (7.9), *n* = 7	1.1–24.2	2.9 (2.6), *n* = 2	1.1–4.7	8.4 (9.0), *n* = 5	2.8–24.2
*Githeri*	7.1 (3.5), *n* = 8	1.1–12.0	7.5 (4.4), *n* = 5	1.1–12.0	6.3 (1.3), *n* = 3	5.3–7.8
*Muthokoi*	6.9 (6.31), *n* = 6	1.0–17.4	7.0 (9.0), *n* = 3	1.0–17.4	6.8 (4.1), *n* = 3	2.1–9.4
All foods	9.0 (7.7), *n* = 41	1.0–32.3	9.2 (8.0), *n* = 19	1.0–29.9	8.8 (7.7), *n* = 22	2.1–32.3

Abbreviations: EBF, exclusive breastfeeding; M. porridge, maize porridge; M. s. porridge, maize‐sorghum porridge; NEBF, nonexclusive breastfeeding.

^a^ *Ugali*: stiff solid maize flour paste.

^b^ *Githeri*: maize grains boiled together with beans.

^c^ *Muthokoi*: dehulled maize boiled together with beans.

#### 3.4.1 Dietary intake of aflatoxin and MOE of lactating mothers in the study

The overall mean dietary intake of total aflatoxin and aflatoxin B1 among lactating mothers was 7.6 μg/kg/b.w.t/day (*SD*, 7.5; range, 0.0–23.9) and 0.6 (*SD*,0.6; range, 0–1.9), respectively (Table 4). Results also showed that the MOE of lactating mothers based on BMDL (0.41 μg/kg/b.w.t/day) against mean and 95th percentile levels of total aflatoxin and aflatoxin B1 dietary intake as shown in Supporting Information: Table S4 were lower than 10,000 cut‐off point adopted by EFSA (2005).

**Table 4:** Dietary intake of aflatoxin among lactating mothers in Kibwezi West.

	Total aflatoxin intake (μg/kg b.w.t/day)					
	All mothers		EBF mothers		NEBF mothers	
Food	x̅ (*SD*)	Range	x̅ (*SD*)	Range	x̅ (*SD*)	Range
Maize *ugali* a	14.4 (6.2), *n* = 14	3.6–23.9	16.7 (6.1), *n* = 8	3.6–23.9	11.2 (5.3), *n* = 6	4.7–17.6
Maize porridge	4.0 (6.2), *n* = 6	0.3–16.1	1.68, *n* = 1	1.68	4.5 (6.7), *n* = 5	0.3–16.1
Maize sorghum porridge	0.1 (0.2), *n* = 7	0–0.5	0 (0), *n* = 2	0–0.0	0.1 (0.2), *n* = 5	0–0.5
*Githeri* b	7.8 (5.3), *n* = 8	0–14.2	6.1 (6.2), *n* = 5	0–14.2	10.7 (1.2), *n* = 3	9.6–12.0
*Muthokoi* c	0.5 (1.3), *n* = 6	0–3.0	1.0 (1.7), *n* = 3	0–3.0	0.0 (0.0), *n* = 3	0.0–0.0
All	7.6 (7.5), *n* = 41	0–23.9	9.4 (8.6), *n* = 19	0–23.9	5.7 (6.4), *n* = 22	0–17.6

	**Aflatoxin B1 intake (μg/kg b.w.t/day)**					
	**All mothers**		**EBF mothers**		**NEBF mothers**	
	**x̅ (** ***SD*** **)**	**Range**	**x̅ (** ***SD*** **)**	**Range**	**x̅ (** ***SD*** **)**	**Range**
Maize ugali	1.2 (0.5), *n* = 14	0.4–1.9	1.0 (0.5), *n* = 8	0.4–1.9	1.4 (0.5), *n* = 6	0.8–1.9
Maize porridge	0.7 (0.4), *n* = 6	0.3–1.1	1.1, *n* = 1	–	0.4 (0.2), *n* = 5	0.3–0.8
Maize sorghum porridge	0.0 (0.1), *n* = 7	0–0.2	0 (0.0), *n* = 2	0 (0.0)	0.0 (0.1), *n* = 5	0–0.2
*Githeri*	0.4 (0.4), *n* = 8	0–1.2	0.5 (0.6), *n* = 5	0.5 (0.6)	0.4 (0.1), *n* = 3	0.3–0.5
*Muthokoi*	0.0 (0.0), *n* = 6	0.0–0.0	0.0 (0.0), *n* = 3	0.0 (0.0)	0.0 (0.0), *n* = 3	0.0–0.0
All	0.6 (0.6), *n* = 41	0–1.9	0.6 (0.6), *n* = 19	0–1.9	0.5 (0.7), *n* = 22	0–1.9

Abbreviations: EBF, exclusive breastfeeding; NEBF, nonexclusive breastfeeding; M. porridge, maize porridge; M. s. porridge, maize‐sorghum porridge.

^a^ *Ugali*: stiff solid maize flour paste.

^b^ *Githeri*: maize grains boiled together with beans.

^c^ *Muthokoi*: dehulled maize boiled together with beans.

### 3.5 Correlation of variables with dietary intake of total aflatoxin and aflatoxin B1 of lactating mothers in Kibwezi West

As shown in Table 5a, a significant negative correlation was reported between the education level of exclusively breastfeeding mothers and the intake of total aflatoxin in the study (*ρ* = −0.47, *p* = 0.040). A negative significant correlation was reported between the dietary diversity of nonexclusively breastfeeding mothers with total aflatoxin intake (*t*_*b*_ = −0.36, *p* = 0.03). Despite cleaning or sorting maize before storage, a positive significant correlation was reported with the intake of total aflatoxin among non‐exclusively breastfeeding mothers (*t*_*b*_ = 0.39, *p* = 0.037). No significant correlation was observed with the remaining variables.

**Table 5a:** Correlation of variables with dietary intake of total aflatoxin of lactating mothers in Kibwezi West.

	Intake of total aflatoxin (μg/kg/b.w.t/day)					
	All mothers (*n* = 41)		EBF mothers (*n* = 19)		NEBF mothers (*n* = 22)	
Variables	*t*_*b*_	*p value*	*t*_*b*_	*p value*	*t*_*b*_	*p value*
*Sociodemographic*						
Age	0.15	0.174	0.27	0.117	0.13	0.435
Education level	−0.27*^ρ^*	0.920	−0.47*^ρ^*	0.040*	−0.43*^ρ^*	0.848
Household size	−0.17	0.154	−0.12	0.512	−0.16	0.347
Socioeconomic status	0.11	0.352	0.08	0.642	0.06	0.706
*Dietary pattern*						
WDD score	−0.02	0.843	0.23	0.225	−0.36	0.030*
Consumption score^a^	0.10	0.407	0.11	0.533	0.17	0.309
*Storage practices*						
Cleaning	0.06	0.643	−0.30	0.140	0.39	0.037*
Applying ash	−0.02	0.854	–	–	0.02	0.908
Chemical treatment	0.07	0.587	0.34	0.094	−0.07	0.720
Drying	−0.05	0.704	−0.26	0.197	0.13	0.486
No treatment	−0.04	0.791	0.10	0.607	−0.30	0.111
*Maize source*						
Market	0.05	0.700	0.03	0.902	0.08	0.659
Shamba	−0.07	0.594	−0.14	0.503	−0.05	0.810
Other source	−0.01	0.916	0.06	0.771	−0.06	0.738
*Place of storage*						
Granary	−0.03	0.836	−0.09	0.652	0.07	0.708
Sacks	−0.07	0.598	−0.08	0.677	−0.08	0.677
Buckets	−0.12	0.379	−0.04	0.825	−0.23	0.217

Abbreviations: EBF, exclusive breastfeeding; NEBF, nonexclusive breastfeeding; ^*ρ*^, Spearman rho correlation; *t*_*b*_, Kendall's tau‐b correlation; WDD, women's dietary diversity.

^a^ consumption score for foods susceptible to aflatoxin contamination (*n* = 38 for all mothers, *n* = 17 for EBF mothers, and *n* = 21 for NEBF mothers.

* Significant at *p* < 0.05.

As shown in Table 5b, socioeconomic status was significantly associated with the intake of aflatoxin B1 among all lactating mothers in the study (*t*_*b*_ = 0.24, *p* = 0.042). Education level was statistically associated with aflatoxin B1 intake among exclusively breastfeeding mothers (*ρ* = −0.56, *p* = 0.012). Household size was statistically correlated to aflatoxin B1 intake among non‐exclusively breastfeeding mothers in the study (*t*_*b*_ = −0.39, *p* = 0.027), while women's dietary diversity score was negatively associated with aflatoxin B1 intake among nonexclusively breastfeeding mothers (*t*_*b*_ = −0.34, *p* = 0.049). However, no significant correlation was observed with the remaining variables.

**Table 5b:** Correlation of variables with dietary intake of aflatoxin B1 of lactating mothers in Kibwezi West.

	Intake of aflatoxin B1 (μg/kg/b.w.t/day)					
	All mothers (*n* = 41)		EBF mothers (*n* = 19)		NEBF mothers (*n* = 22)	
Variables	*t*_*b*_	*p value*	*t*_*b*_	*p value*	*t*_*b*_	*p value*
*Social demographic*						
Age	0.13	0.249	0.26	0.135	0.08	0.623
Education level	−0.10*^ρ^*	0.529	−0.56*^ρ^*	0.012*	−0.43*^ρ^*	0.848
Household size	−0.21	0.084	−0.07	0.689	−0.39	0.027*
Socioeconomic status	0.24	0.042*	0.30	0.086	0.18	0.282
*Dietary pattern*						
WDD score	−0.08	0.491	0.14	0.462	−0.34	0.049*
Consumption score^a^	0.01	0.919	0.02	0.901	0.09	0.600
*Storage practices*						
Cleaning	0.06	0.672	−0.24	0.228	0.34	0.072
Applying ash	0.12	0.391	–	–	0.13	0.486
Chemical treatment	0.02	0.861	0.16	0.421	−0.07	0.720
Drying	0.04	0.780	−0.26	0.197	0.26	0.164
No treatment	−0.08	0.560	0.19	0.350	−0.35	0.061
*Maize source*						
Market	0.08	0.576	0.02	0.934	0.13	0.476
Shamba	−0.04	0.780	0.00	–	−0.11	0.575
Other source	−0.06	0.682	−0.02	0.934	−0.09	0.639
*Place of storage*						
Granary	0.00	0.987	−0.02	0.910	0.00	1.000
Sacks	−0.09	0.499	0.14	0.479	−0.22	0.238
Buckets	−0.09	0.503	−0.15	0.452	−0.07	0.720

Abbreviations: EBF, exclusive breastfeeding; NEBF, nonexclusive breastfeeding; ^*ρ*^, Spearman rho correlation; *t_b_*, Kendall's tau‐b correlation; WDD, women's dietary diversity.

^a^ consumption score for foods susceptible to aflatoxin contamination (*n* = 38 for all mothers, *n* = 17 for EBF mothers, and *n* = 21 for NEBF mothers.

* Significant at *p* < 0.05.

### 3.6 Predictors of aflatoxin exposure of lactating mothers of children 0–6 months

Among predictor variables, as shown in Table 6, the level of education was found to significantly and negatively influence estimates of aflatoxin B1 intake among exclusively lactating mothers in the study (*β* = −0.56, *p* = 0.01). Women's dietary diversity scores were found to negatively influence estimates of aflatoxin B1 intake among nonexclusively lactating mothers (*β* = −0.43, *p* = 0.04). The regression coefficient using the @Risk simulation model showed maize ugali as the greatest contributor to cumulative intake of both total aflatoxins (*b* = 0.69) and aflatoxin B1 intake (*b* = 0.70). The least contributor to the cumulative intake of both total aflatoxin and aflatoxin B1, however, was reported for ‘muthokoi’ (*b* = 0.02) (Supporting Information: Figure S1).

**Table 6:** Predictors of aflatoxin exposure among lactating mothers breastfeeding children 0–6 months in Kibwezi West.

	Unstandardised coefficients		Standardised coefficients		
Predictors	*B*	Std. Error	*β*	*t*	Sig.
*AFT conc (NEBF mothers)*					
Household size	27.68	13.43	0.42	2.06	0.052
*AFT intake (NEBF mothers)*					
Women dietary diversity score	−1.35	1.05	−0.27	−1.28	0.22
Cleaning maize before storage	6.55	3.85	0.36	1.70	0.11
*AFB1 intake (All mothers)*					
Socioeconomic status	0.06	0.031	0.296	1.938	0.06
*AFB1 intake (EBF mothers)*					
Mother level of education	−0.23	0.08	−0.56	−2.81	0.01*
*AFB1 intake (NEBF mothers)*					
Household size	−0.25	0.13	−0.38	−1.93	0.69
Women dietary diversity score	−0.22	0.10	−0.43	−2.21	0.04*

Abbreviations: AFT, total aflatoxin; AFB1, aflatoxin B1; EBF, exclusively breastfeeding; NEBF, nonexclusively breastfeeding.

* Significant at *p* < 0.05.

## 4 DISCUSSION

The observed high mean household size of lactating mothers compared to the national mean was expected as rural areas in Kenya are generally characterised by larger household sizes (Kenya Population and Housing Census [KPHC], 2019b). The majority of mothers interviewed were multiparous mothers of whom studies, including that of Mohamed et al. (2018), have associated with exclusive and prolonged breastfeeding. The number of children per lactating mother, however, was similar to those reported in Makueni County (mean of 3.3) (KDHS, 2014). The percentage of lactating mothers who had attained basic education was lower than the national rate (67.3%) (KDHS, 2014). This was indicative of low educational status among lactating mothers in the study area. However, the age demography of lactating mothers was similar to those reported by KDHS (2014). Low to modest wealth index, income levels and occupation status reported are pointers of low economic status in the study area. Consumption expenditure of less than a dollar per day by almost 60% indicates low disposable income in the study area. This might be one of the factors that deter mothers from accessing quality and diverse diets in the study area. However, most socioeconomic and demographic variables between exclusively and non‐exclusively lactating mothers were similar. This was expected as the study area population is almost homogenous.

According to weekly consumption frequency, ‘ugali’, maize porridge, ‘githeri’ and groundnut would put lactating mothers at a higher risk of aflatoxin exposure. This did not come as a surprise as these foods constitute staple foods mostly consumed in Kenya. Other foods that could easily contribute to aflatoxin exposure include animal milk (mostly cow's milk) in the form of milk tea and ‘muthokoi’. However, their frequency of consumption was modest per week in the study. As fish, chicken, cassava, finger millet, eggs, plain sorghum flour and mixed flour porridge were rarely consumed per week in the study, they were considered to contribute less to dietary aflatoxin exposure among lactating mothers. Overall, the food frequency result of this study was similar to those of Kilonzo et al. (2014) in Makueni, and Magoha et al. (2014) in Tanzania. However, an aflatoxin consumption score of <25% out of 18 foods shows that dietary exposure in the study area likely comes from a limited range of foods. This outcome is in parallel with the results of this study which showed low consumption frequency of foods that are least susceptible to aflatoxin contamination. In fact, out of 16 foods listed in the food frequency questionnaire, kale, cowpea leaves, cabbage, beans, and pigeon peas were the most consumed on a weekly basis. Comparatively, the 3.4 mean women's dietary diversity score reported in this study was lower than the 5.5, and 4.2 mean scores reported in Tanzania (Magoha et al., 2014) and Nepal (Andrews‐Trevino et al., 2020), respectively. From these results, it is clear that the dietary diversity of lactating mothers in this study is considerably low, and is similar to those of Nabwire et al. (2022) in Makueni. These results are indicative of low food availability and accessibility in the study area. The low socioeconomic status reported in this study is a pointer that low purchasing power could be a barrier to meeting adequate dietary diversity and quality food in the area. However, it has also been noted that the hot harsh climate does not favour agricultural activities in this study area.

Based on consumption quantities, the mean consumption of maize ‘ugali*’* (340.5 g/day) and maize porridge (412.3 g/day) were higher than 195.5 and 38.6 g/day, respectively, reported by Kilonzo et al. (2014) in Makueni. However, estimates of ‘githeri’ (93.6 g/day) and ‘muthokoi’ (36.2 g/day) in this study were comparable to the estimates of the same study by Kilonzo et al. (2014) (‘githeri’ [103.3 g/day] and ‘muthokoi’ [28.6 g/day]). Consumption of maize ‘ugali’ was again similar to 360 g/day reported by Kang'ethe et al. (2017) also in Makueni. Conversely, consumption of animal milk (150 g/day) and rice (250 g/day) among lactating mothers in northern India was higher than those reported in this study (19.5 and 30.6 g/day, respectively). Similarly, the consumption of eggs in this study (1.7 g/day) was lower than those reported among mothers in Iran (50 g/day) (Azarikia et al., 2018). From these results, it is evident that maize‐based foods constitute a larger portion of mothers' dietary intake in the study region than other foods. Finally, the absence of significant differences in food frequency, dietary diversity and consumption quantities between breastfeeding groups shows that all lactating mothers in the study area have similar food consumption patterns.

This study agrees with Koskei et al. (2020) and Malusha et al. (2016) that metal and plastic buckets, sacks and granaries are the commonly used maize storage containers in Makueni. Okoth and Ohingo (2004) further showed that storage in plastics, polythene bags, metal buckets and sacks leads to a moisture content of at least 13.6% in maize. In the environment of a hot and humid temperature, it is highly possible that storage practices could be a cause of the high prevalence of aflatoxin in Makueni. Just as the study of Nii et al. (2019), the storage of maize products for a long time in these containers could exacerbate the occurrence of aflatoxin in the area. The findings of this study showed that most households in Makueni bought maize from the market, followed by their cultivation and other sources (gifts/donations/reliefs). These findings were similar to the ones reported by Daniel et al. (2011). However, varying results linking aflatoxin contamination and source have been reported in the study area. For instance, the study by Daniel et al. (2011) showed that home‐produced maize was highly contaminated by aflatoxin compared to the ones bought from the market and those given out as relief food. On the other hand, Mwihia et al. (2008) showed that maize produced at home and those bought from the market were all highly contaminated with aflatoxin. This study, however, did not categorise the food samples into sources, and, thus, could not link the source of maize and aflatoxin occurrence in the study. Lastly, the proportion of lactating mothers applying methods that could reduce aflatoxin occurrence in maize was found to be lower than those reported by Koskei et al. (2020). It is thus clear that maize handling and storage practices among lactating mothers are still low to mitigate the occurrence of aflatoxin in the study area.

The prevalence of 85.4% of aflatoxin in the study was generally as high as those reported by Nabwire et al. (2020) (100%) and Kang'ethe et al. (2017) (80.4%) in Makueni. This high prevalence confirms the suspicion of this study about the existence of prolonged and frequent occurrence of aflatoxin contamination in the area. The prevalence of this present study with that of Kilonzo et al. (2014) (45%, 20% and 35% for maize kernels, ‘muthokoi’ and maize meal samples, respectively) in Makueni, points out that cooked maize dishes are also a source of dietary aflatoxin exposure in the area. Studies like the one of Obonyo and Salano (2018) and Nabwire et al. (2020) reported a high prevalence of aflatoxin in raw maize. However, due to the handling and processing of maize before cooking, the prevalence in this study was expected to be at least moderate. This result could suggest that maize handling and processing practices in the study area are not effective enough in reducing contamination of aflatoxin in raw maize before cooking or milling. Similarly, aflatoxin concentrations were expected to be lower than those reported for raw maize; however, this was not the case. The mean concentration of over 90% of food samples was shown to exceed the 2, and 10 µg/kg set limits for aflatoxin B1 and total aflatoxin, respectively. The mean of total aflatoxin (97.8 µg/kg) in this study was higher than 62.5 µg/kg of Nabwire et al. (2020) and 41.5 µg/kg of Kang'ethe et al. (2017) in Makueni. They were, however, within the ranges of 6–480 µg/kg reported by Kilonzo et al. (2014) in the same study area. These results reaffirm earlier statements about the high occurrence of aflatoxin contamination and possible prolonged aflatoxin exposure in the study area. They also explain why the likelihood of consuming foods contaminated with aflatoxin between exclusively and nonexclusively lactating mothers reported in the study was comparable. The results further support the findings that imply that once aflatoxins are inside the food matrix, they can withstand cooking temperatures without getting destroyed. However, the mean concentration level of aflatoxin B1 (9.0 μg/kg) in this study was not as high as those reported for total aflatoxin. This was also affirmed when the result of this present study was compared against the mean levels of aflatoxin B1 reported by Mahuku et al. (2019) (39.0 μg/kg) and Nabwire et al. (2020) (59.3 μg/kg) in Makueni County. The mismatch between levels of total aflatoxin and aflatoxin B1 in this study is in agreement with the findings of Matumba et al. (2015). In this experiment, it was concluded that aflatoxin ratios within the food matrix will always vary between those reported for total aflatoxin and individual types of aflatoxin. However, the high proportion of food samples exceeding the KEBS limit is alarming and should be a cause of food safety concerns in the study area.

The mean dietary intake of total aflatoxin (7600 ng/kg b.w.t/day, i.e., converted from μg/kg b.w.t/day) among lactating mothers in this study was remarkably higher than those reported by Kilonzo et al. (2014) (27.23, 291.66 and 59.31 ng/kg b.w.t/day for ‘muthokoi’, maize kernel and maize meal, respectively). They were also higher than those reported by Kang'ethe et al. (2017) (260 ng/kg b.w.t/day) in Makueni. Equally, the results were higher than 271 ng/kg b.w.t/day in Ghana (Kortei et al., 2019) and 1.29 ng/kg b.w.t/day in Turkey (Kabak, 2021). Mean intake of aflatoxin B1 (600 ng/kg b.w.t/day, that is, converted from μg/kg b.w.t/day), on the other side, was comparatively higher than 451.8 ng/kg b.w.t/day reported in Makueni and 148.4 ng/kg b.w.t/day reported in the western region of Kenya (Mahuku et al., 2019). Additionally, the exposure ranges of aflatoxin B1 dietary intake in this study (0–1900 ng/kg b.w.t/day, i.e., converted from μg/kg b.w.t/day) were wider than the ranges of aflatoxin B1 estimated for Kenya adults (35–133 ng/kg b.w.t/day) (Liu & Wu, 2010), and surpassed the upper bound exposure levels of aflatoxin B1 intake of 3.25 ng/kg b.w.t/day estimated for adults from several studies (EFSA CONTAM Panel et al., 2020). The levels were equally higher than the 1.19 ng/kg b.w.t/day reported in Ghana (Kabak, 2021). The high intake levels reported in this study could be because this present study also factored in the consumption frequency of foods within a day. However, going by these results, it is evident that dietary intake of both total aflatoxin and aflatoxin B1 is higher among lactating mothers in the study area. The high intake was greatly attributed to the high consumption of maize ‘ugali’ followed by maize porridge, maize sorghum porridge, ‘githeri’ and ‘muthokoi’ in that order. This outcome, however, did not come as a surprise because maize ugali is the main staple food in Kenya, and is most frequently consumed among other maize‐based foods. Kilonzo et al. (2014) findings in Makueni were also similar to the results of this study.

However, the absence of a significant association between dietary intake levels of aflatoxin and the breastfeeding status of lactating mothers reaffirms the earlier stated view that exclusively and non‐exclusively breastfeeding mothers in the study area are at equal risk of being predisposed to dietary aflatoxin. The calculated MOE values in this present study were less than one (<1), and this was interpreted as extremely low compared to the results of other studies. For instance, Marijani et al. (2020) in Kenya reported a higher MOE value of 126.3 from the consumption of ‘omena’ (*Rastrineola argentea*). Kabak (2021), on the other hand, reported a margin exposure value of 336 in Ghana. The extremely low MOE values reported in this study is indicative that lactating mothers in Kibwezi and its surrounding could be at higher risk of carcinogenic exposure compared to other areas. As a result of this, risk characterisation is highly recommended among lactating mothers and breastfeeding children in the study area.

Though the predictor model showed that household size was not a significant influencer of total aflatoxin in the study, the positive significant correlation between them may suggest that larger households in the study area are at higher risk of consuming aflatoxin‐contaminated maize. As maize is the main staple food in Kenya and is consumed by almost everyone, its demand per person is hypothesised to be higher in larger households. This demand is matched by stocking for larger quantities of maize or frequently sourcing in smaller amounts from different sources. Either of these practices, combined with poor handling and storage reported among lactating mothers in the study, could increase the chances of maize being contaminated with aflatoxin in larger households. This hypothesis is in agreement with Nabwire et al. (2022), who found that children from smaller size families in Makueni had lower aflatoxin exposure as opposed to their counterparts.

Further, the model containing both women's dietary diversity and cleaning of maize before storage response was found to be a major predictor of dietary intake of total aflatoxin among lactating mothers. Even though the *p* values for the two variables were not significant in the model, a significant negative correlation of dietary diversity of lactating mothers found in the study area concurs with the observation made by Nabwire et al. (2022), who showed that low dietary diversity in Makueni was associated with increased risk of aflatoxin intake. On the other hand, cleaning maize before storage in this study was found not to influence the exposure of lactating mothers to aflatoxins. This observation, however, was not expected as the study by Lesuuda et al. (2021) concluded that separating deformed grains was associated with a low occurrence of aflatoxins. This could therefore mean that the process is not effective enough to reduce a substantial amount of aflatoxin once in the maize food samples. The results could also be indicative of high levels of aflatoxin contamination in the study area as compared to others in Kenya. For other practices, no significant relationship was reported between concentration levels of total aflatoxin, and aflatoxin B1 in the analysed foods with maize source, handling, processing and storage practices in the study. These results were different from those reported by Nabwire et al. (2020) and Daniel et al. (2011), among others, who linked aflatoxin contamination with maize source, handling, processing and storage practices in Makueni. These differences could be because the result of this study was based on mothers' responses, while the latter was based on experimental analysis.

Higher regression coefficients reported in this study also point out that frequency of intake, food type and quantities of foods consumed play an important role in determining the exposure levels of dietary aflatoxin intake among lactating mothers in the study. Therefore, it was not surprising to associate higher dietary intake of aflatoxin in the study area with consumption of maize ugali > maize porridge > maize sorghum porridge > ‘githeri’ > ‘muthokoi’ in that order. Just as earlier results of this present study, education was also shown to reduce aflatoxin B1 intake by about 45% among exclusively lactating mothers. This result supports the findings of Leroy et al. (2015) but contrasts with the findings of Mehta et al. (2021). That notwithstanding, findings shared by Malusha et al. (2016) and Lesuuda et al. (2021) showed a negative correlation between knowledge, attitude and practices with aflatoxin contamination on cereals, and underscores education as one of the strategies that can be implemented among lactating mothers to reduce dietary aflatoxin exposure in this present study. Though the socioeconomic status model did not predict aflatoxin B1 intake of lactating mothers in the study, its positive association with an increase in aflatoxin B1 intake was not expected. This is because the results of Leroy et al. (2015), Nabwire et al. (2020) and a review by Omara et al. (2021), among others, had associated households with lower socioeconomic index with a higher probability of aflatoxin exposure within the same study area of this present study. Similarly, this study showed that almost 95% of lactating mothers in the study were below the upper wealth index. Further investigation for a possible explanation was conducted. Results of this study though nonsignificant, showed a positive association between socioeconomic status and an increase in the total number of meals consumed per day by lactating mothers and an increase in women's dietary diversity in the study. But a predictor model showed that women dietary diversity negatively influenced aflatoxin B1 intake among non‐exclusively lactating mothers. This outcome which showed that low dietary diversity was a risk factor for aflatoxin intake, was also consistent with those reported by Leroy et al. (2015) and Nabwire et al. (2022) conducted in the same area of this study but inconsistent with those reported away from this study area by Mehta et al. (2021) in India. However, when focusing on foods that are only susceptible to aflatoxin contamination, the study by Andrews‐Trevino et al. (2020) reported a positive correlation between socioeconomic status and consumption frequency of contaminated maize and groundnuts among Nepalese women. This study, therefore, suggests that the influence of socioeconomic status on aflatoxin levels in foods depends on the region of the study, the available type and range of food diversity and the prevalence of aflatoxin contamination in the area. When used on its own, it might not be a reliable pointer of aflatoxin intake in a study area. For instance, without basic knowledge of aflatoxin contamination and limited food choice, lactating mothers with higher socioeconomic status can still be susceptible to aflatoxin B1 exposure in the diet. Even though Andrews‐Trevino et al. (2020) reported a negative association between age and exposure to aflatoxin B1 in the serum of pregnant mothers, this study, regardless of the breastfeeding status, did not find any direct influence of age of the lactating mothers on the total aflatoxin and aflatoxin B1 levels in the study area. This could probably be due to a range of sociodemographic and economic similarities drawn between exclusively and nonexclusively lactating mothers in the study.

## 5 CONCLUSION

The high prevalence and presence of aflatoxin in foods of lactating mothers are a public health concern and calls for the need to devise easy‐to‐use household food safety and monitoring measures in the study area. However, health practitioners, policymakers and food safety experts should also scale awareness among lactating mothers, and small ‐and ‐medium‐scale maize and cereals enterprises in the study area about aflatoxin and its associated short and long‐term impacts on humans. Additionally, information regarding aflatoxins and mothers' diets should be integrated into nutrition education materials in the study region as a way of recommending adherence to dietary practices that ensure food safety among mothers during lactation. Results also show that improving socioeconomic status, education levels and access to food could reduce the levels of dietary aflatoxin exposure among lactating mothers in the study area.

## AUTHOR CONTRIBUTIONS

Isaac O. Ogallo designed the research study, sought ethical clearance, conducted data collection, performed the statistical analysis, and drafted the research paper. Alice M. Mwangi supervised and offered technical guidance in designing the research study, financially assisted in the acquisition of experiment kits for laboratory analysis, and reviewed the research paper. Dasel W. M. Kaindi and George O. Abong supervised, edited, and offered technical guidance in drafting the research paper and data analysis.

## CONFLICT OF INTEREST STATEMENT

The authors declare no conflicts of interest.

## Supporting information

Supporting information.

## Data Availability

Data available on request due to privacy/ethical restrictions.

## Footnotes

3 Dietary diversity score food groups used in this study were as per FAO (2011) at the time of data collection. It was therefore not possible to use the newly adopted Minimum Dietary Diversity for Women as per FAO and FHI 360 (2016) guidelines.
