# Supplementary material for: Dietary aflatoxin exposure of lactating mothers of children 0–6 months in Makueni County, Kenya
Source: Matern Child Nutr. 2023 Feb 22;19(3):e13493. doi: 10.1111/mcn.13493 (PMC10262888; doi:10.1111/mcn.13493)
Supplement: Supplementary file 1 — Supporting information. [file MCN-19-e13493-s001.docx]

**Figures and supporting materials**

Supplementary Table S1: Consumption frequency per week of foods likely to be contaminated with aflatoxins in Kibwezi West

|  | | **% Frequency per week EBF ^a^ (n=75), NEBF ^b^ (n=95), all (n=170)** | | | | | | | | |  | | |
| --- | --- | --- | --- | --- | --- | --- | --- | --- | --- | --- | --- | --- | --- |
| **Food** |  | | **1** | **2** | **3** | **4** | **5** | **6** | **7** | **Rarely** | | | **χ^2^** |
| Maize *ugali*^†^ | ***EBF*** | | 5.3 | 4.0 | 20.0 | 14.7 | 8.0 | 13.3 | 34.7 | - | | 0.15 | |
|  | ***NEBF*** | | 0.0 | 2.1 | 14.7 | 16.8 | 13.7 | 8.4 | 44.2 | - | | - | |
|  | ***All*** | | 2.4 | 2.9 | 17.1 | 15.9 | 11.2 | 10.6 | 40.0 | - | | - | |
| Maize porridge | ***EBF*** | | 9.3 | 4.0 | 21.3 | 18.7 | 14.7 | 10.7 | 21.3 | - | | 0.66 | |
|  | ***NEBF*** | | 3.2 | 5.3 | 23.2 | 21.1 | 14.7 | 15.8 | 16.8 | - | | - | |
|  | ***All*** | | 5.9 | 4.7 | 22.4 | 20.0 | 14.7 | 13.5 | 18.8 | - | | - | |
| Sorghum | ***EBF*** | | 2.7 | 5.3 | 1.3 | 6.7 | 1.3 | 4.0 | 1.3 | 77.3 | | 0.03^*^ | |
|  | ***NEBF*** | | 10.5 | 2.1 | 3.2 | 2.1 | 0.0 | 0.0 | 5.3 | 76.8 | | - | |
|  | ***All*** | | 7.1 | 3.5 | 2.4 | 4.1 | 0.6 | 1.8 | 3.5 | 77.1 | | - | |
| Mixed porridge | ***EBF*** | | 1.3 | 2.7 | 2.7 | 4.0 | 2.7 | 1.3 | - | 85.3 | | 0.94 | |
|  | ***NEBF*** | | 0.0 | 2.1 | 3.2 | 2.1 | 3.2 | 3.2 | - | 86.3 | | - | |
|  | ***All*** | | 0.6 | 2.4 | 2.9 | 2.9 | 2.9 | 2.4 | - | 85.9 | | - | |
| *Githeri* ^‡^ | ***EBF*** | | 29.3 | 20.0 | 20.0 | 4.0 | 2.7 | 2.7 | 5.3 | 16.0 | | 0.80 | |
|  | ***NEBF*** | | 34.7 | 24.2 | 14.7 | 5.3 | 5.3 | 2.1 | 4.2 | 9.5 | | - | |
|  | ***All*** | | 32.4 | 22.4 | 17.1 | 4.7 | 4.1 | 2.4 | 4.7 | 12.4 | |  | |
| *Muthokoi* ^§^ | ***EBF*** | | 26.7 | 10.7 | 5.3 | 2.7 | 2.7 | 1.3 | - | 50.7 | | 0.59 | |
|  | ***NEBF*** | | 27.4 | 13.7 | 9.5 | 2.1 | 0.0 | 0.0 | - | 47.4 | | - | |
|  | ***All*** | | 27.1 | 12.4 | 7.6 | 2.4 | 1.2 | 0.6 | - | 48.8 | | - | |
| Rice | ***EBF*** | | 28.0 | 18.7 | 8.0 | 2.7 | 2.7 | 0.0 | - | 40.0 | | 0.11 | |
|  | ***NEBF*** | | 31.6 | 7.4 | 4.2 | 1.1 | 1.1 | 1.1 | - | 53.7 | | - | |
|  | ***All*** | | 30.0 | 12.4 | 5.9 | 1.8 | 1.8 | 0.6 | - | 47.6 | | - | |
| Finger millet | ***EBF*** | | 4.0 | 1.3 | 1.3 | 1.3 | - | - | 4.0 | 88.0 | | 0.18 | |
|  | ***NEBF*** | | 3.2 | 2.1 | 0.0 | 0.0 | - | - | 0.0 | 94.7 | | - | |
|  | ***All*** | | 3.5 | 1.8 | 0.6 | 0.6 | - | - | 1.8 | 91.8 | | - | |
| Cassava | ***EBF*** | | 16.0 | 2.7 | 1.3 | 1.3 | 1.3 | - | - | 77.3 | | 0.91 | |
|  | ***NEBF*** | | 15.8 | 1.1 | 2.1 | 1.1 | 0.0 | - | - | 80.0 | | - | |
|  | ***All*** | | 15.9 | 1.8 | 1.8 | 1.2 | 0.6 | - | - | 78.8 | | - | |
| Groundnut | ***EBF*** | | 24.0 | 28.0 | 12.0 | 10.7 | 8.0 | 8.0 | - | 9.3 | | 0.01^*^ | |
|  | ***NEBF*** | | 26.3 | 18.9 | 8.4 | 0.0 | 20.0 | 12.6 | - | 13.7 | | - | |
|  | ***All*** | | 25.3 | 22.9 | 10.0 | 4.7 | 14.7 | 10.6 | - | 11.8 | | - | |
| Beef | ***EBF*** | | 30.7 | 4.0 | 2.7 | 5.3 | 2.7 | - | - | 54.7 | | 0.12 | |
|  | ***NEBF*** | | 31.6 | 7.4 | 2.1 | 0.0 | 0.0 | - | - | 58.9 | | - | |
|  | ***All*** | | 31.2 | 5.9 | 2.4 | 2.4 | 1.2 | - | - | 57.1 | | - | |
| Chicken | ***EBF*** | | 22.7 | 1.3 | 0.0 | 0.0 | - | - | - | 76.0 | | 0.96 | |
|  | ***NEBF*** | | 18.9 | 2.1 | 1.1 | 1.1 | - | - | - | 76.8 | | - | |
|  | ***All*** | | 20.6 | 1.8 | 0.6 | 0.6 | - | - | - | 76.5 | | - | |
| Eggs | ***EBF*** | | 30.7 | 4.0 | 1.3 | 0.0 | - | - | - | 64.0 | | 0.60 | |
|  | ***NEBF*** | | 22.1 | 7.4 | 2.1 | 1.1 | - | - | - | 67.4 | | - | |
|  | ***All*** | | 25.9 | 5.9 | 1.8 | 0.6 | - | - | - | 65.9 | | - | |
| Fish | ***EBF*** | | 13.3 | 1.3 | - | - | - | - | - | 85.3 | | 0.03^*^ | |
|  | ***NEBF*** | | 4.2 | 0.0 | - | - | - | - | - | 95.8 | | - | |
|  | ***All*** | | 8.2 | 0.6 | - | - | - | - | - | 91.2 | | - | |
| Milk | ***EBF*** | | 26.7 | 6.7 | 4.0 | 4.0 | 5.3 | 0.0 | 10.7 | 42.7 | | 0.88 | |
|  | ***NEBF*** | | 28.4 | 5.3 | 5.3 | 5.3 | 3.2 | 1.1 | 5.3 | 46.3 | | - | |
|  | ***All*** | | 27.6 | 5.9 | 4.7 | 4.7 | 4.1 | 0.6 | 7.6 | 44.7 | | - | |

EBF: exclusive breastfeeding mothers, NEBF: non-exclusive breastfeeding mothers

-: not reported

^†^*Ugali:* stiff solid maize flour paste.

^‡^ *Githeri*: maize grains boiled together with beans.

^§^*Muthokoi*: dehulled maize boiled together with beans.

*Significant at p < 0.05

Supplementary Table S2: Consumption frequency per week of foods unlikely to be contaminated with aflatoxin in Kibwezi West

| **% Frequency per week EBF (n=75), NEBF (n=95), all (n=170)** | | | | | | | | | | |
| --- | --- | --- | --- | --- | --- | --- | --- | --- | --- | --- |
| **Food** |  | **1** | **2** | **3** | **4** | **5** | **6** | **7** | **Rarely** | **χ^2^** |
| Irish potatoes | *EBF* | 17.3 | 13.3 | 4.0 | - | - | - | - | 65.3 | 0.18 |
|  | *NEBF* | 12.6 | 5.3 | 3.2 | - | - | - | - | 78.9 | - |
|  | *All* | 14.7 | 8.8 | 3.5 | - | - | - | - | 72.9 | - |
| Sweet potatoes | *EBF* | 13.3 | 1.3 | 100 | - | - | - | - | 85.3 | 0.76 |
|  | *NEB* | 4.2 | 3.2 | 100 | - | - | - | - | 92.6 | - |
|  | *All* | 8.2 | 2.4 | 100 | - | - | - | - | 89.4 | - |
| Bananas | *EBF* | 16.0 | 8.0 | 1.3 | - | - | - | - | 74.7 | 0.72 |
|  | *NEBF* | 10.5 | 7.4 | 1.1 | - | - | - | - | 81.1 | - |
|  | *All* | 12.9 | 7.6 | 1.2 | - | - | - | - | 78.2 | - |
| Pawpaw | *EBF* | 6.7 | - | - | - | - | - | - | 93.3 | 0.86 |
|  | *NEBF* | 7.4 | - | - | - | - | - | - | 92.6 | - |
|  | *All* | 7.1 | - | - | - | - | - | - | 92.9 | - |
| Mangoes | *EBF* | 21.3 | 16.0 | 5.3 | 2.7 | 4.0 | - | - | 50.7 | 0.97 |
|  | *NEBF* | 20.0 | 13.7 | 6.3 | 3.2 | 2.1 | - | - | 54.7 | - |
|  | *All* | 20.6 | 14.7 | 5.9 | 2.9 | 2.9 | - | - | 52.9 | - |
| Oranges | *EBF* | 24.0 | 9.3 | 5.3 | 1.3 | - | - | - | 60.0 | 0.78 |
|  | *NEBF* | 22.1 | 7.4 | 2.1 | 2.1 | - | - | - | 66.3 | - |
|  | *All* | 22.9 | 8.2 | 3.5 | 1.8 | - | - | - | 63.5 | - |
| Kales | *EBF* | 8.0 | 16.0 | 12.0 | 13.3 | 22.7 | 10.7 | 10.7 | 6.7 | 0.94 |
|  | *NEBF* | 6.3 | 15.8 | 14.7 | 20.0 | 18.9 | 9.5 | 7.4 | 7.4 | - |
|  | *All* | 7.1 | 15.9 | 13.5 | 17.1 | 20.6 | 10 | 8.8 | 7.1 | - |
| Cabbage | *EBF* | 25.3 | 10.7 | 2.7 | 5.3 | 4.0 | 1.3 | 5.3 | 45.3 | 0.17 |
|  | *NEBF* | 28.4 | 11.6 | 10.5 | 4.2 | 6.3 | 1.1 | 0.0 | 37.9 | - |
|  | *All* | 27.1 | 11.2 | 7.1 | 4.7 | 5.3 | 1.2 | 2.4 | 41.2 | - |
| Carrots | *EBF* | 18.7 | 2.7 | 0.0 | 0.0 | - | - | - | 78.7 | 0.49 |
|  | *NEBF* | 11.6 | 3.2 | 2.1 | 1.1 | - | - | - | 82.1 | - |
|  | *All* | 14.7 | 2.9 | 1.2 | 0.6 | - | - | - | 80.6 | - |
| Cow pea leaves | *EBF* | 8.0 | 2.7 | 5.3 | 13.3 | 16.0 | 10.7 | 4.0 | 40.0 | 0.58 |
|  | *NEBF* | 10.5 | 4.2 | 8.4 | 16.8 | 15.8 | 6.3 | 9.5 | 28.4 | - |
|  | *All* | 9.4 | 3.5 | 7.1 | 15.3 | 15.9 | 8.2 | 7.1 | 33.5 | - |
| Amaranth | *EBF* | 8.0 | 2.7 | 2.7 | 2.7 | 1.3 | - | - | 82.7 | 0.23 |
|  | *NEBF* | 10.5 | 10.5 | 6.3 | 3.2 | 2.1 | - | - | 67.4 | - |
|  | *All* | 9.4 | 7.1 | 4.7 | 2.9 | 1.8 | - | - | 74.1 | - |
| *Managu*^†^ | *EBF* | 9.3 | 5.3 | 4.0 | 2.7 | - | - | - | 78.7 | 0.95 |
|  | *NEBF* | 11.6 | 6.3 | 2.1 | 3.2 | - | - | - | 76.8 | - |
|  | *All* | 10.6 | 5.9 | 2.9 | 2.9 | - | - | - | 77.6 | - |
| Beans | *EBF* | 34.7 | 17.3 | 6.7 | 8.0 | 5.3 | - | - | 28.0 | 0.72 |
|  | *NEBF* | 30.5 | 16.8 | 14.7 | 7.4 | 4.2 | - | - | 26.3 | - |
|  | *All* | 32.4 | 17.1 | 11.2 | 7.6 | 4.7 | - | - | 27.1 | - |
| Pigeon peas | *EBF* | 6.7 | 1.3 | 1.3 | 5.3 | 4.0 | - | - | 81.3 | 0.45 |
|  | *NEBF* | 9.5 | 6.3 | 1.1 | 4.2 | 1.1 | - | - | 77.9 | - |
|  | *All* | 8.2 | 4.1 | 1.2 | 4.7 | 2.4 | - | - | 79.4 | - |
| Green grams | *EBF* | 9.3 | 12.0 | 8.0 | 2.7 | 0.0 | - | - | 68.0 | 0.09 |
|  | *NEBF* | 18.9 | 4.2 | 4.2 | 1.1 | 2.1 | - | - | 69.5 | - |
|  | *All* | 14.7 | 7.6 | 5.9 | 1.8 | 1.2 | - | - | 68.8 | - |

EBF: exclusive breastfeeding mothers, NEBF: non-exclusive breastfeeding mothers

^†^ *Managu*: (*Solanum nigrum*)

-: not reported

*Significant at p < 0.05

**Supplementary Table S3: Weekly aflatoxin consumption score of lactating mothers in Kibwezi West**

|  | **All**  **(N=170)** | | **EBF**  **(n=95)** | | | **NEBF**  **(n=75)** | | **M-W *U-*test**  **EBF*NEBF** | |
| --- | --- | --- | --- | --- | --- | --- | --- | --- | --- |
| **Food** | **x̅ (*SD*)** | **Range** | | **x̅ (*SD*)** | **Range** | **x̅ (*SD*)** | **Range** | | ***p*-value** |
| Sorghum *ugali*^†^ | 1.2(3.7) | 0-28 | 1.1(3.9) | | 0-28 | 1.3(3.6) | 0-21 | | 0.55 |
| Maize *ugali* | 9.4(6.3) | 1-28 | 7.8 (5.0) | | 1-28 | 10.7(7.1) | 2-28 | | 0.01^*^ |
| Mixed *ugali* | 0.9(2.5) | 0-14 | 0.6(1.8) | | 0-7 | 1.1(2.9) | 0-14 | | 0.48 |
| *Githeri* ^‡^ | 3.6(3.9) | 0-21 | 3.2(3.5) | | 0-21 | 3.8 (4.1) | 0-21 | | 0.31 |
| *Muthokoi*^§^ | 1.3(1.8) | 0-8 | 1.3 (1.8) | | 0-8 | 1.4 (1.8) | 0-8 | | 0.87 |
| Maize porridge | 9.2(5.1) | 1-24 | 9.0 (5.3) | | 1-21 | 9.4 (5.0) | 1-24 | | 0.49 |
| Mixed porridge | 1.5(4.1) | 0-21 | 1.3 (3.5) | | 0-16 | 1.7(4.6) | 0-21 | | 0.99 |
| Cassava porridge | 0.8(3.5) | 0-24 | 1.4 (4.6) | | 0-24 | 0.4(2.4) | 0-20 | | 0.16 |
| Finger millet | 0.4(2.3) | 0-21 | 0.8(3.3) | | 0-21 | 0.1(0.6) | 0-6 | | 0.10 |
| Groundnuts | 5.0(4.8) | 0-20 | 4.5(4.2) | | 0-20 | 5.3(5.3) | 0-20 | | 0.80 |
| Sorghum porridge | 1.4(3.2) | 0-18 | 1.6 (3.7) | | 0-18 | 1.1 (2.8) | 0-14 | | 0.87 |
| Rice | 1.4(2.2) | 0-15 | 1.8 (2.5) | | 0-15 | 1.1 (1.9) | 0-12 | | 0.02^*^ |
| Cassava tuber | 0.5(1.2) | 0-8 | 0.5 (1.4) | | 0-8 | 0.5 (1.1) | 0-6 | | 0.84 |
| Beef | 0.8(1.2) | 0-6 | 0.9(1.4) | | 0-6 | 0.7(0.9) | 0-4 | | 0.42 |
| Chicken | 0.3(0.6) | 0-4 | 0.3 (0.5) | | 0-2 | 0.3 (0.7) | 0-4 | | 0.99 |
| Eggs | 0.6(1.3) | 0-9 | 0.5(1.2) | | 0-6 | 0.7(1.4) | 0-9 | | 0.88 |
| Fish | 0.1(0.3) | 0-2 | 0.1(0.4) | | 0-2 | 0.04(0.2) | 0-1 | | 0.02^*^ |
| Dairy Milk | 2.1(3.5) | 1-21 | 2.3(3.6) | | 0-15 | 2.0(3.4) | 0-21 | | 0.65 |

EBF: exclusive breastfeeding mothers, NEBF: non-exclusive breastfeeding mothers, M-W: Mann Whitney

^†^*Ugali:* stiff solid maize flour paste.

^‡^ *Githeri*: maize grains boiled together with beans.

^§^*Muthokoi*: dehulled maize boiled together with beans.

*Significant at p < 0.05

Supplementary Table S4: Margin of exposure to dietary aflatoxin among lactating mothers in Kibwezi West

|  | **AFT Intake**  **(μg/kg b.w.t/day)** | **MOE** | **AFB1**  **(μg/kg b.w.t/day)** | **MOE** |
| --- | --- | --- | --- | --- |
| *All lactating mothers (n=41)* |  |  |  |  |
| Mean | 7.6 | 0.05 | 0.6 | 0.68 |
| p95 | 20.9 | 0.02 | 1.9 | 0.22 |
|  |  | |  | |
| *EBF ^e^ mothers (n=19)* | |  |  |  |
| Mean | 9.4 | 0.04 | 0.6 | 0.68 |
| p95 | 23.5 | 0.02 | 1.9 | 0.22 |
|  |  |  |  |  |
| *NEBF ^f^ mothers (n=22)* |  |  |  |  |
| Mean | 5.6 | 0.07 | 0.5 | 0.82 |
| p95 | 17.4 | 0.02 | 1.9 | 0.21 |

EBF: Exclusive breastfeeding; NEBF: Non-exclusive breastfeeding; AFT: Total aflatoxin; AFB1: Aflatoxin B1; MOE: Margin of exposure based on mean and 95^th^ percentile (p95) of aflatoxin dietary intake

Supplementary Figure S1: Regression coefficient of dietary aflatoxin intake of each food on cumulative intake of aflatoxin (μg/kg/b.w.t/day) among lactating mothers in Kibwezi West

**Appendix 1: Food frequency questionnaire showing the list of the foods, their frequencies, and corresponding food scores in Kibwezi West**

| **Food Consumption Pattern for Lactating Mothers (15-45 Years)** | | | | | | | | | | | | |
| --- | --- | --- | --- | --- | --- | --- | --- | --- | --- | --- | --- | --- |
| **Food** | **Indicate consumption Frequency (per week) and estimate the amount in grams** | | | | | | | | **After every two weeks** | **Monthly** | **Never** | **Food Scores Daily=7**  **Once=1**  **Twice=2**  **Thrice=3**  **Four times=4**  **Five times=5**  **Six times=6**  **Per Fortnight=0**  **Per Month=0 Never=0** |
|  | **Once/week** | **Twice/week** | **Thrice/week** | **Four/week** | **Five/week** | **Six/week** | **Daily** | **Number of intakes within a typical day** |  |  |  |  |
| **cereals, roots, tubers, and protein sources** | | | | | | | | | | | | |
| Maize meal (Ugali) |  |  |  |  |  |  |  |  |  |  |  | * |
| Sorghum flour ugali |  |  |  |  |  |  |  |  |  |  |  | * |
| Mixed flour ugali |  |  |  |  |  |  |  |  |  |  |  | * |
| *Githeri* |  |  |  |  |  |  |  |  |  |  |  | * |
| *Muthokoi* |  |  |  |  |  |  |  |  |  |  |  | * |
| Maize flour porridge |  |  |  |  |  |  |  |  |  |  |  | * |
| Sorghum flour porridge |  |  |  |  |  |  |  |  |  |  |  | * |
| Mixed flour porridge |  |  |  |  |  |  |  |  |  |  |  | * |
| Cassava flour porridge |  |  |  |  |  |  |  |  |  |  |  | * |
| Sorghum flour porridge |  |  |  |  |  |  |  |  |  |  |  | * |
| Rice |  |  |  |  |  |  |  |  |  |  |  | * |
| Cassava |  |  |  |  |  |  |  |  |  |  |  | * |
| Finger millet |  |  |  |  |  |  |  |  |  |  |  | * |
| Beef |  |  |  |  |  |  |  |  |  |  |  | * |
| Chicken |  |  |  |  |  |  |  |  |  |  |  | * |
| Egg |  |  |  |  |  |  |  |  |  |  |  | * |
| Fish |  |  |  |  |  |  |  |  |  |  |  | * |
| Milk |  |  |  |  |  |  |  |  |  |  |  | * |
| Food score percent | | | | | | | | | | | | (Total food score/504 × 100%) |
